# Obese Animals as Models for Numerous Diseases: Advantages and Applications

**DOI:** 10.3390/medicina57050399

**Published:** 2021-04-21

**Authors:** Abdelaziz Ghanemi, Mayumi Yoshioka, Jonny St-Amand

**Affiliations:** 1 Department of Molecular Medicine, Faculty of Medicine, Laval University, Québec, QC G1V 0A6, Canada; abdelaziz.ghanemi@crchudequebec.ulaval.ca; 2 Functional Genomics Laboratory, Endocrinology and Nephrology Axis, CHU de Québec-Université Laval Research Center, 2705 Boul. Laurier, Québec, QC G1V 4G2, Canada; mayumi.yoshioka@crchudequebec.ulaval.ca

**Keywords:** obese, animal, model, disease, obesity, pathogenesis

## Abstract

With the advances in obesity research, a variety of animal models have been developed to investigate obesity pathogenesis, development, therapies and complications. Such obese animals would not only allow us to explore obesity but would also represent models to study diseases and conditions that develop with obesity or where obesity represents a risk factor. Indeed, obese subjects, as well as animal models of obesity, develop pathologies such as cardiovascular diseases, diabetes, inflammation and metabolic disorders. Therefore, obese animals would represent models for numerous diseases. Although those diseases can be induced in animals by chemicals or drugs without obesity development, having them developed as consequences of obesity has numerous advantages. These advantages include mimicking natural pathogenesis processes, using diversity in obesity models (diet, animal species) to study the related variabilities and exploring disease intensity and reversibility depending on obesity development and treatments. Importantly, therapeutic implications and pharmacological tests represent key advantages too. On the other hand, obesity prevalence is continuously increasing, and, therefore, the likelihood of having a patient suffering simultaneously from obesity and a particular disease is increasing. Thus, studying diverse diseases in obese animals (either induced naturally or developed) would allow researchers to build a library of data related to the patterns or specificities of obese patients within the context of pathologies. This may lead to a new branch of medicine specifically dedicated to the diseases and care of obese patients, similar to geriatric medicine, which focuses on the elderly population.

Obesity remains one of the most challenging health problems worldwide, with increasing prevalence [1]. It impacts both public health and the economy [2], and it has even worsened within the ongoing context of COVID-19 [3]. In health science, obesity is defined based on body mass index (BMI), which represents a clinical measure of body composition using weight over height-squared [4,5]. Obesity involves abnormal fat accumulation, initiated with adipocyte expansion via numerous underlying pathways as a result of an unhealthy lifestyle pattern (diet, physical inactivity, sleeping) combined with genetic factors, microbiota and psychology [6]. Obesity has even been hypothesized to have neuroendocrine reprogramming [7] that would make obesity hard to “reverse” once established. The medical danger of obesity is not limited to the increased body weight nor the social and psychological impacts on life quality [8]. In fact, the comorbidities and risks for health associated with obesity represent important and relevant medical problems [6]. Indeed, obesity is associated with a variety of diseases and health disorders. These include heart and cardiovascular diseases [9,10], inflammation [11,12], respiratory disease [13,14], stroke [15,16], diabetes [17,18], psychological disorders such as depression [19,20], iron deficiency [21], bone disorders [22] and even neurodegenerative diseases [23,24,25], cancer [26,27] and regeneration impairment [28]. In addition, the metabolically active tissues, including liver and adipose tissue and skeletal muscle, are also impacted during obesity. Indeed, obesity impacts on the liver can include portal inflammation, fibrosis and cirrhosis [29]. In a key tissue of energy expenditure, modified metabolic properties of muscle during obesity [30] can contribute to the development of sarcopenic obesity [31] and accumulate triacylglycerol in the lipid droplets that cause a lipotoxicity state [32]. Regarding adipose tissue, which is the main energy storage tissue, lipids are stored following the conversion of extra glucose into triglycerides [32]. Such impacts on these metabolically active tissues would worsen obesity-induced metabolic imbalances and lead to further metabolic disorders, including mitochondrial dysfunction [30] and insulin resistance [33], among other manifestations of metabolic syndrome during obesity [34]. Since obesity itself represents a status of imbalanced energy metabolism following broken homeostasis with a modified neuroendocrine profile [6,7], impacts on the main metabolic tissues worsen the metabolic phenotype. Other important changes are seen via the disturbed pattern of bioactive molecules produced by these metabolic tissues during obesity, including hormones, growth factors, cytokines and other adipokines [35] that impact a variety of tissues and biologically functional homeostasis. Within this context, whether to classify obesity as a disease represents a hot topic, with diverse medical and legal consequences [36,37,38,39,40]. 

Therefore, it is urgent to deepen our biological understanding of obesity. Within this perspective, different animal models have been developed through approaches aiming to induce obesity within a determined period of time. Once the obesity status is established, a variety of measures are conducted in order to explore diverse patterns, including obesity pathogenesis, obesity development and antiobesity therapy efficacy. These measures could be with or without exogenous interventions, including diet control, physical exercise and bariatric surgery.

Most importantly, the development and prognosis of obesity-related comorbidities and associated health problems such as strokes, metabolic disorders, lipid profile abnormalities, inflammations and cancer are key elements to explore as well. These animal models aim to mimic the obesity status and allow the exploration of the related clinical and therapeutic outcomes along with their implications and applications. 

On the other hand, an obese animal could develop (depending on numerous factors) one or more obesity-associated diseases and disorders. In this obese animal, those diseases and disorders are considered biological consequences or comorbidities associated with its obesity status. Therefore, obese animals would represent “natural models of choice” compared to the known obesity animal models to study the different aspects of those health problems using "naturally" conditioned animals over "artificially" induced ones. Below, we briefly expose the key reasons beyond such a statement.

**In vivo mimicking of obesity-induced comorbidities and changes**: Obesity is associated with various comorbidities and biological consequences. These same comorbidities can also be induced by exogenous treatments in addition to being the result of obesity. For instance, rather than inducing disease-like inflammation via lipopolysaccharides [41] or administering streptozotocin to induce diabetes [42], we would rather have these diseases “naturally” triggered by obesity and developed in obese animals. Compared to such chemically generated models of these conditions (inflammation and diabetes induced by lipopolysaccharides and streptozotocin, respectively, in these examples), obese animals are models that could develop these same conditions but in a naturally occurring manner. Importantly, the process occurs through pathogenic pathways similar to the in vivo mechanisms. Therefore, obese animals have the advantages of not only mimicking the status and the symptoms but also mimicking the biological processes leading to these outcomes and comorbidities (e.g., inflammation, diabetes) within obesity. This makes these models (obese animals) more “natural”. Indeed, the conditions would be the result of molecular and cellular processes caused by the obesity-generated environment, representing biochemically broken homeostasis [6,28] rather than an exogenous intervention (“artificial” model). Indeed, the “artificial” models lead only to the same final outcome but without all the underlying pathways. Therefore, we will miss the pathogenic patterns and links by only studying the comorbidities dissociated from obesity. 

**Diversity of animal models**: Many animal models exist for obesity [43,44], and they vary in both species and the method of inducing obesity. The availability of different animals (e.g., mouse [45,46], rat [47], pig [48], rabbit [49]), allows diverse animal species choices for the development of an obese model. The choice is based on factors including genetics, size, life expectancy and generation time. This diversity facilitates the study of each obesity-related condition (e.g., brain modifications [50], epigenetics [51], impacts on pregnancy [52], metabolic phenotype [53]) in the most suitable animals. For instance, large mammals such as pigs would be closer to humans for studying the impacts of bariatric surgery due to size, whereas mice, also used to study bariatric surgeries [54], would be more suitable to study maternal diet impact on offspring due to the short generation time as well as the important litter size. However, in many cases, financial reasons (animal cost) and the available animal care facilities would limit the choices and explain the wide usage of mice models rather than pigs, for example. The usage of more expensive models (for instance, pigs rather than mice, as well as monkeys) would be justified by practical design, including anatomical or physiological similarities (and fat cell size) to humans [55] or specific genetic patterns within some defined contexts, such as pathogenesis.

Some of the used approaches to generate obese animals in different studies are diet (mainly high-fat diet) [56,57,58,59], modified genes such as ob/ob [60,61] and db/db mice [62,63], and castration-induced obesity [64,65]. Even within the same method, variety can exist. For instance, diet-induced obesity is among the most widely used. However, the type of diet used in different studies [66,67,68,69,70] could be different from one model to another in terms of percentage of lipids, protein and carbohydrate as well as type of lipids, total calories and carbohydrate source. Therefore, these lead to diverse biological and pathological outcomes, especially where there is no standard protocol [71]. Having many choices is of particular importance when we want to (1) build an obesity model reflecting the diet of a specific region or population in order to mimic the obesity patterns that are developed by that population, and, therefore, (2) study geography-specific or population-specific obesity patterns, such as the Western-style diet [72]. Within the context of diet, the role of the hypothalamus in the pathogenesis of obesity, pointed out even before leptin discovery [73], would contribute to the regulation of energy balance signals [74,75,76]. Indeed, the hypothalamus, particularly in rodent models of diet-induced obesity, is impacted by obesity and plays an important role in the pathogenesis of obesity [74,77,78,79,80]. This has critical importance for the development of neuropharmacological therapies for obesity [81,82] involving the hypothalamus [83,84,85,86].

This animal model diversity does significantly extend the possible choices in terms of animals, animal strains and types of diet. However, it remains important to mention the drawbacks to this diversity of animal models. For instance, there are the issues of whether we can make comparisons across studies and also the possible limited reproducibility (even when two studies use the same high-fat diet, would both use the same standard chow diet as a control?). This means that studies with similar designs can have different outcomes due to differences in animal strain, age, and diet content. Moreover, some studies use only male animals, neglecting to include both sexes, which represents another drawback that also requires optimizations towards standard parameters to further include sex- and gender-related variations. In addition, different designs might lead to different findings based on animal choice (e.g., genetics, age). Furthermore, it is also critical to consider the impacts of parameters external to the animal, such as temperature, housing conditions, and sacrifice time. Importantly, it is also worth stating that obesity impacts social and psychological well-being [87]. Such parameters might be hard to evaluate in animal models, which are among the limitations that animal models of obesity have. Differences between humans and mice represent the basis of most disadvantages and limitations of such models, which includes the need for months of a high-fat diet to produce the full symptoms of type 2 diabetes mellitus [71]. This is why a struggle persists when extrapolating the results from animal studies to clinical applications.

**Disease intensity and reversibility:** Following the initial line of thought, presenting obesity as the inducer of specific diseases, it seems acceptable to say that disease developmental stage and severity scale are dependent on obesity intensity [88]. Therefore, we can compare the development of conditions such as inflammation, prediabetes and even psychological consequences [89] in obese animals based on different obesity intensity measured by selected parameters such as lipid percentage, body fat [90,91] and body weight [4]. Importantly, the ability to reverse obesity [92,93]—at least a short-term reversion by reducing body weight—would allow us to observe how the other diseases and disorders evolve. Most importantly, we would observe whether those diseases and disorders are also reversible once obesity is “treated”. For instance, we can monitor diabetes or inflammation [94] marker changes following obesity treatment, which would be of great importance from clinical and therapeutic perspectives. Moreover, these changes may also be recorded depending on the approaches used to treat or reverse obesity (e.g., diet, physical activity [95], pharmacology [96], bariatric surgery [97]). This could be a methodology to evaluate and compare the different therapeutic approaches and add new evaluation and clinical tools for research. Importantly, this will allow us to establish pathological and mechanistic links between obesity and its associated comorbidities, which are among the most important missing patterns that limit our molecular and cellular understating of obesity.

**Monitoring therapeutic implications**: We can target—with antiobesity therapy—one disease among those existing in obese animals (such as diabetes) and see how the other related disease prognoses (such as stroke) evolve. This goes beyond therapeutic evaluation and could point to the chronological order of appearance of the different obesity-related disorders. Herein, it can also show whether a disorder (such as inflammation [98]) is a direct consequence of obesity or the indirect result of one of the diseases induced by obesity. This can be clarified by targeting the conditions of a pathway and then monitoring another condition to find out whether this second condition belongs to or is impacted by the same pathway as the first one. This will elucidate the mechanistic pathways and pathogenesis in chronological order. Recent molecular data characterizing gene activation in the context of obesity as well as diet and exercise [99,100,101,102,103,104] will also help the mapping of the chronology of such pathways, ultimately building bridges between gene activation and obesity-related comorbidities through identified molecular and cellular pathways in chronological order. Illustrative examples of obese animals that produce disease or health condition models have been reported in the literature (Table 1). This illustrates how complex health conditions and biological consequences of obesity can be explored in obese animals. Therefore, these animals would be considered models for these specific health conditions or diseases as well.

Expanding the explored conditions to cover the remaining obesity-related comorbidities in different obese animals will complete the puzzle of both obesity-induced disease pathogenesis and the underlying mechanistic steps. Importantly, diseases such as cancer and COVID-19 include obesity among their risk factors (rather than causes) [26,114,115]. Therefore, an explorative comparison of obese animals that develop a disease such as cancer and the obese animals that do not could allow a better understanding of the mechanisms that convert a risk factor into a cause. This requires investigating key patterns of metabolism, physiology, gene expression and diet in both animal groups. This can, for instance, solve some mysteries beyond cancer, one of the leading causes of death [116,117] that is related to obesity. Among the emerging obesity-related research fields, microbiota has great potential for both understanding obesity pathogenesis and developing innovative therapeutic approaches [118]. Diet, an important factor in both obesity development and the building of obesity animal models, can modify the microbiota composition [119]. In addition, obese subjects have been reported to have a microbiota composition different from that seen in lean subjects [120]. Microbiota composition may modify the metabolism and energy balance of the host [67] via pathways suggested to either lead to obesity [67,120] or positively improve the metabolic profile [120]. Thus, scientists are exploring the use of fecal (microbiota) transplants as well as probiotics as therapies to improve obesity, among other diseases [121,122,123,124,125,126,127], through beneficially modifying microbiota composition. Importantly, we can also consider obese animals as models of dysbiosis since this microbiota composition change occurs naturally as a consequence of obesity.

These perspectives, presenting obese animals as models for a variety of diseases, could be extrapolated to other conditions that—like obesity—have multiple comorbidities such as diabetes [128,129,130,131]. Furthermore, obese animals can be used as starting models to build more complex models by inducing some disorders (such as infections) in obese animals and observing how a disease would evolve or how therapy efficacy would be affected in an obesity-induced environment compared to a nonobese environment. Such studied control groups would be both obese animals not suffering from that disease (or not receiving the treatment) and nonobese animals suffering from that same disease (or receiving the treatment). Such experimental design will allow us to explore obesity as well as the induced disease or the applied treatment as variables and investigate their interactions. For clinical (and preclinical aspects), the properties linking obesity to its related diseases, explored in obese animals, can be extrapolated to humans, within the known limits [132], during clinical studies. Obesity prevalence [133] is continuously increasing. Thus, the likelihood of having a patient that is simultaneously suffering from obesity and another disease increases accordingly, especially during these times of the COVID-19 epidemic [3]. Therefore, studies within this context, supported by the results obtained from obese animals, as described above, would allow researchers to build a library of data related to the patterns or specificities of obese patients within the context of pathologies. Importantly, we look forward to a new branch of medicine that deals with the diseases and care of obese patients, similar to geriatric medicine, which focuses on the elderly population. 

## Figures and Tables

**Table 1:** Examples of obese mice that are used or could be used as disease/health condition models.

Developed Disease (Models)	Obesity-Induction and Animal Models	References
Peripheral diabetic neuropathy	ob/ob mice	[105]
Hepatic steatosis	ob/ob mice	[106]
Type 2 diabetes and diabetic retinal neurodegeneration	db/db mice	[107,108]
Type 2 diabetes	ob/ob mice	[109]
Abnormal gut microbiota composition	db/db mice	[110]
Steatohepatitis	foz/foz mice fed a high-fat diet	[111]
Inflammation	Mice fed a high-fat diet	[112,113]
Insulin resistance	Mice fed a high-fat diet	[113]

## Data Availability

Not applicable.
